# Non-Surgical Treatment of Biliary Affections

**Published:** 1908-06

**Authors:** 


### ABSTRACT.

### Non-Surgical Treatment of Biliary Affections

Ludwig Woelfert, Brooklyn, N. Y., (*International Journal of Surgery*, March, 1908), says there is a class of cases which shows typical symptoms of cholelithiasis, without colic and in which differential diagnosis from simple cholangitis cannot be made with absolute certainty. There is perhaps a small but steady absorption of biliary secretion into the circulation, and occasional exacerbations manifest themselves by jaundice. A corresponding insufficiency or deficiency in the quantity or quality of bile causes a continuous disturbance of the digestive tract. Constipation exists, and there may be diarrhea from excessive putrefaction, consequent destruction of erythrocytes, with anemia and its train of symptoms—neuralgias, headaches, visual disturbances, dizziness, emaciation. The bile absorbed into the circulation facilitates diapedesis of the erythrocytes through the capillaries and may even cause their complete emigration. There may be capillary hemorrhage from any mucous surface, and it may even be subcutaneous.

The one common diagnostic feature is that the urine exhibits abnormal contents of chromogens and of indoxyl and skatoxyl derivatives. The following test which he has devised and which is sharp, reliable and quickly made, will be found useful:

Four to five c.c. of urine are mixed with an equal volume of hydrochloric acid; a dark red or purplish color reaction indicates an excess of skatoxyl. About one c.c. of chloroform and onehalf to two drops of liq. sod. chlorin., U.S.P., are then added, and the mixture is shaken for a few seconds; the appearance of the blue reaction of the chloroform, which may vary from the least tinge to the deepest indigo, shows indican. Upon heating the indigo is gradually converted into its isomeric red modification, the chloroform thereby changing from blue to purple and red. This change of color is also seen with other indican tests, the rapidity with which it occurs depending much on temperature and strength of reagent. Excess of liq. sod. chlorin. discolors the chloroform. In the supernatant liquid the nature of the chromogen chromophore may be observed by its color, which may be anything from yellow to black. The study of the significance of these various colors offers a field for further investigation. Of course, medicines taken by the patient may modify the color reactions.

Excess of indoxyl and skatoxyl does not, per se, indicate disturbances of the liver or its appendages; there may be gastric dilatation, cancer or ulcer, affections of the pancreas, duodenum or ileum, mixed tubercular infection, or suppurative processes anywhere. Skatoxyl and its congeners predominate in affections of the large intestine. But when associated with the subjective difficulties enumerated above, biliary disease can be diagnosticated with certainty.

Catarrh in the biliary system on the basis of bacterial invasion and stasis of the portal circulation, furnishes medical indications. Presence of stones does not necessarily demand operative procedure; stones are often found at autopsies of subjects who never had the least symptom of biliary trouble. Appropriate medicinal means can often place stones into a state of harmless latency, or possibly put the viscus into a condition to eliminate them, by stimulating the formation of so much thin bile that the stream becomes powerful enough to expel the calculi, their mobilisation being favored by reestablishing normal conditions in the ducts.

He resorts to the usual dietetic and hygienic measures and to the use of biliary antiseptics and stimulants and analeptics. That a combination of salicylic acid, sodium oleate, phenolphthalein and menthol is effective, he knows from his experience with the probilin pills of Bauermeister. He uses them whenever the urinary test gives positive result, including hepatic and gastrointestinal cancer—for symptomatic benefit—and anemia due to bile absorption. He has just discharged, after three months' use of the pills, a very obstinate and marked case with symptoms of choleic disorders dating back as far as twenty years. She had for a number of years drifted from one specialist to another, her normal ovaries having been finally removed without relief. She took 2 pills twice daily with hot water, and later 4 pills twice daily. At the time of writing she is greatly improved, free from distress, and the anemia is yielding, now that normal bile flow is restored.

